# Effects of senescent secretory phenotype acquisition on human retinal pigment epithelial stem cells

**DOI:** 10.18632/aging.101624

**Published:** 2018-11-16

**Authors:** Raffaella Lazzarini, Michele Nicolai, Vittorio Pirani, Cesare Mariotti, Roberto Di Primio

**Affiliations:** 1 Department of Clinical and Molecular Sciences-Histology, Università Politecnica delle Marche, Ancona 60020, Italy; 2 Ophthalmology Clinic, Università Politecnica delle Marche, Ancona 60020, Italy; * Equal contribution

**Keywords:** AMD, RPESCs, age-related diseases, senescence, inflammation

## Abstract

Regenerative medicine approaches based on mesenchymal stem cells (MSCs) are being investigated to treat several aging-associated diseases, including age-related macular degeneration (AMD). Loss of retinal pigment epithelium (RPE) cells occurs early in AMD, and their transplant has the potential to slow disease progression.

The human RPE contains a subpopulation of cells - adult RPE stem cells (RPESCs) – that are capable of self-renewal and of differentiating into RPE cells *in vitro*. However, age-related MSC changes involve loss of function and acquisition of a senescence-associated secretory phenotype (SASP), which can contribute to the maintenance of a chronic state of low-grade inflammation in tissues and organs.

In a previous study we isolated, characterized, and differentiated RPESCs. Here, we induced replicative senescence in RPESCs and tested their acquisition of the senescence phenotype and the SASP as well as the differentiation ability of young and senescent RPESCs.

Senescent RPESCs showed a significantly reduced proliferation ability, high senescence-associated β-galactosidase activity, and SASP acquisition. RPE-specific genes were downregulated and p21 and p53 protein expression was upregulated.

These findings document the effects of senescence and SASP acquisition on RPESC differentiation ability and highlight the need for a greater understanding of their role in AMD pathogenesis.

## Introduction

Age-related macular degeneration (AMD) is an eye disorder affecting the elderly which can induce an irreversible loss of central visual function [1]. Its limited treatment possibilities and the rising number of senior citizens in developed countries are reasons for concern; indeed, it has been estimated that 200 million individuals will suffer from AMD in 2020 and almost 300 million will be affected in 2040 [2]. Aging plays a major role in AMD pathogenesis [3]; smoking, cataract surgery, a high body mass index (BMI), a history of cardiovascular disease, and a family history of AMD are additional risk factors [4,5].

The Age-Related Eye Disease Study (AREDS) has classified AMD into early, intermediate, and late stage [6], while the Clinical Age-Related Maculopathy Staging (CARMS) system divides patients into five mutually exclusive categories based on slit-lamp assessment of clinical features [7]. However, there is no consensus on the staging and progression terminology. In 2013, the Beckman Initiative for Macular Research Classification Committee proposed a new clinical classification based on the three AREDS stages: stage 1, normal, aging phenotype (small drusen < 63 μm without pigmentary changes); stage 2, early AMD with medium drusen (63 - 125 μm) and no pigmentary abnormalities; and stage 3, which is subdivided into intermediate – large drusen and/or pigmentary changes – and advanced – choroidal neovascularization (CNV, exudative or neovascular AMD) or geographic atrophy (GA; dry or non-exudative AMD) [8]. Anti-vascular endothelial growth factor (anti-VEGF) has long been the mainstay of treatment for neovascular AMD, whereas no effective treatment is available for the more common dry form [9–12].

AMD is the result of complex interactions among metabolic, functional, genetic, and environmental factors [13]. It is characterized by degeneration of the retinal pigment epithelium (RPE), death of photoreceptors and degradation of choriocapillaries, which together lead to impairment and loss of central vision. The RPE is a polarized non-proliferative cell monolayer lying between the neural retina and the vascularized choroid. It serves several functions that are essential for vision and for the survival of retinal neurons. Its dysfunction, due to oxidative stress, mitochondrial destabilization, and complement dysregulation, has been implicated in AMD pathogenesis [14,15]. During aging, RPE cells undergo a number of functional alterations that result in the development of age-related eye disorders, including AMD. RPE cell damage induced by proinflammatory factors – including tumor necrosis factor (TNF)-α – and cell death are able to promote activation of the alternative pathway (AP) of the complement system at the retina-choroid interface, a process that has been associated to RPE cell death; aging also induces a reduction in the number and an increase in the size and multinucleation of RPE cells [16]. The activation of different protein kinase C isoforms has also been implicated in the age-related formation of multinucleated RPE cells [17].

Neovascular AMD and GA are characterized by RPE dysfunction [18]; in GA, formation of large confluent drusen and hyperpigmentation (presumably related to the RPE dysfunction) seem to be the initial insult, while drusen resorption and RPE loss (hypopigmentation) are believed to predict its progression. Photoreceptor and choriocapillary dysfunction and death appear to be secondary to RPE loss; loss of choriocapillaries with an intact RPE has also been described in wet AMD [18]. According to a recent study, RPE cells from AMD patients show a different phenotype as well as functional changes such as altered autophagy, mitochondrial dysfunction, and susceptibility to oxidative stress compared to those from normal individuals [19].

The early and intermediate stages of AMD are characterized by changes in lipid metabolism, autophagy, and inflammation; molecular signaling pathways, such as inflammation, cellular senescence and cell death, play a key role in the progression of late-stage dry AMD, whereas angiogenesis predominates in the neovascular form [3]. A greater understanding of the molecular pathways that are involved in the various stages of AMD would contribute to the development of innovative therapies.

An imbalance of circulating inflammatory molecules seems to characterize most age-related diseases (ARDs). Aging is characterized by a state of chronic, low-grade inflammation, known as inflammaging [20], which also appears to be involved in all stages of AMD development and progression.

Senescent cells are non-proliferating cells capable of secreting proinflammatory cytokines, thus contributing to ARD development and ARD-related morbidity. Cellular senescence is characterized by cell growth arrest, altered DNA synthesis and repair, resistance to apoptosis, and increased cell size [21]. Telomere shortening, DNA damage, and oxidative stress are capable of activating senescence processes [22].

Mesenchymal stem cells (MSCs) have been isolated from different adult tissues, including the RPE [23]. Ease of isolation, high proliferation potential, and low immunogenicity make them ideal for cell-based therapies. MSC function declines with age; senescent MSCs acquire a senescence-associated secretory phenotype (SASP) that contributes to driving aging and the development and progression of ARDs, including AMD [24].

The human RPE contains a subpopulation of stem-like MSCs (RPESCs) [23]. In a previous work, we isolated and characterized human RPESCs, demonstrated their ability to differentiate into mesenchymal (adipogenic, osteogenic and chondrogenic) lineages, and analyzed their differentiation potential into neuronal and retinal lineages [25]. In a recent study of an AMD rat model, transplantation of RPESCs isolated from human RPE was able to prevent visual loss [26].

The mechanisms involved in the activation of differentiation of resident RPESCs into mature RPE cells and the role of RPESCs in AMD pathogenesis are still unclear. A growing number of studies have been addressing the role of persistent inflammation in AMD development and progression. The aim of this work is explore the molecular mechanisms that are involved in the acquisition of the senescent phenotype by RPESCs and the role of its proinflammatory factors in altering the function of aged RPE cells. To do so, we investigated the characteristics of replicative and stress-induced senescence of RPESCs, their morphological and genetic features, and their acquisition of the SASP, all of which seem to play a role in AMD pathogenesis.

## RESULTS

### Replicative senescence of human RPESCs

RPESCs were isolated, characterized, and cultured as described in a previous work by our group [25]. They were isolated from a healthy eye from a 21-year-old donor. All experiments were conducted using 3 different batches of exponentially growing cells at the 3^rd^ passage (confluence about 75%) in which replicative senescence was induced.

**Figure 1:**
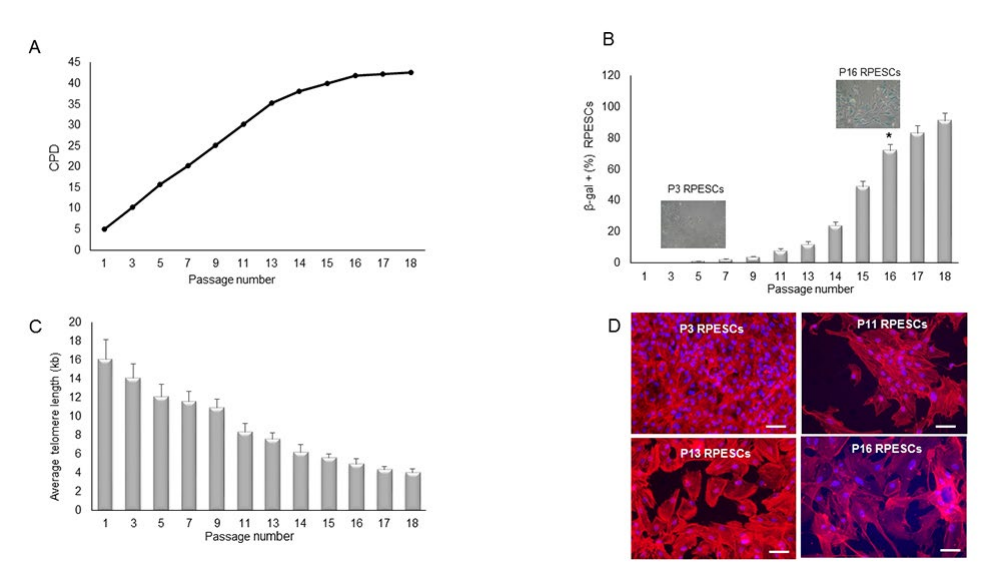
**Proliferation rate, β-gal positivity, telomere length, and cell morphology during RPESC replicative senescence.** RPESC replicative senescence. (**A**) Cumulative number of population doublings (CPD) in RPESCs grown to senescence. (**B**) Percentage of β-gal-positive cells detected during RPESC replicative senescence from P1 to P16. P11, number of culture passages. Data are reported as mean ± SD. *P =0.039. (**C**) RPESC telomere length during replicative senescence was analyzed from P1 to P18; data are reported as mean ± SD of 3 independent experiments. (**D**) Morphological analysis of young (P3) and senescent (P16) RPESCs by the TRIC-phalloidin immunofluorescence assay. Senescent RPESCs appear enlarged and flattened. Magnification 20X, scale bar 200 µm. Pictures are representative of 3 independent experiments.

Replicative senescence was documented at the 16^th^ passage (P16) by arrested proliferation, increased β-galactosidase (SA β-gal) activity, and telomere length reduction. In particular, RPESC proliferation rate increased steeply from P2 to P11 and plateaued at P13; growth stopped at P18 (Figure 1A). As illustrated in Figure 1B, the proportion of β-gal-positive cells increased significantly (*P* < 0.05) from 6 ± 0.04% in young cells (P3-P6) to 80 ± 12.1% in senescent cells (P15-P18). Telomere length declined from 14 kb at P3 to 4 kb at P16 (Figure 1C). TRIC-phalloidin staining demonstrated that senescent RPESCs had a flatter and enlarged morphology compared to young cells (Figure 1D).

### Acquisition of the secretory phenotype by senescent RPESCs

SASP acquisition by senescent REPSCs was determined by analyzing a panel of proinflammatory molecules: interleukin (IL)-6, interferon (INF)-γ, TNF-α, IL-12, and transforming growth factor β (TGFβ)1 - in young (P3) and senescent (P16) RPESCs. The analysis was extended by determination of the levels of the anti-inflammatory molecules IL-4, IL-10, and IL-13.

**Figure 2:**
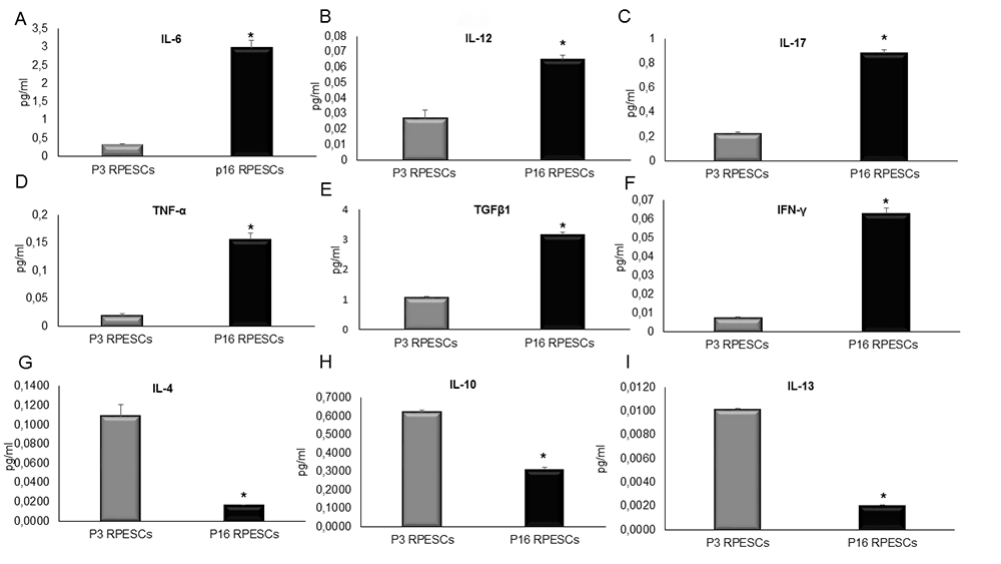
**SASP induction in senescent RPESCs.** Young (P3) and senescent (P16) RPESCs were maintained in culture for 48 h. The supernatant was analyzed for IL-6 (**A**), IL-12 (**B**), IL-17 (**C**), TNF-α (**D**), TGFβ1 (**E**), INF-γ (**F**), IL-4 (**G**), IL-10 (**H**), and IL-13 (**I**), by ELISA. Data are mean ± SD of 3 independent experiments. *P = from 0.021 to 0.041.

The results of ELISA indicated that senescent RPESCs secreted higher levels of IL-6, IL-12, IL-17, TNF-α, TGFβ1, and INF-γ compared to young RPESCs (Figure 2A-F), reflecting a proinflammatory state and SASP acquisition. They also secreted lower levels of several anti-inflammatory molecules, including IL-4, IL-10, and IL-13, compared to young RPESCs (Figure 2G-I).

### Expression of stemness, reprogramming, and RPE-specific genes in young and senescent RPESCs

The expression of RPE-specific (*OTX2, PEDF, PAX6, RPE65, MITF,* and *CRALBP*), stemness, and reprogramming (*SOX2, KLF4* and *c-MYC*) genes was investigated in young and senescent RPECS by qRT-PCR.

**Figure 3:**
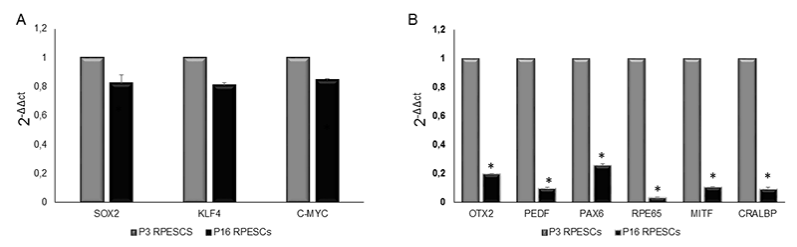
**mRNA expression levels of stemness and RPE-specific genes in senescent and young RPESCs.** (**A**) qRT-PCR analysis of the expression levels of stemness genes in senescent (P16) and young (P3) RPESCs. (**B**) qRT-PCR analysis of the mRNA levels of RPE-specific genes in senescent and young RPESCs. Data are mean ± SD of 3 independent experiments. *P = from 0.026 to 0.036.

As shown in Figure 3A, the two sets of cells showed similar expression levels of *SOX2, KLF4* and *c-MYC*, whereas senescent RPESCs exhibited downregulation of *OTX2, PEDF, PAX6, RPE65, MITF,* and *CRALBP* (Figure 3B).

### Determination of the mRNA levels of senescence-associated genes in senescent and young RPESCs by PCR Array

The expression of senescence-associated genes in senescent and young RPESCs was measured by analyzing their mRNA levels using Cellular Senescence RT2 Profiler PCR Array (PAHS, Qiagen). Glyceraldehyde 3-phosphate dehydrogenase (*GAPDH*) and ribosomal protein, large, P0 (*RPLP0*) were used as inner controls to normalize gene expression levels.

**Figure 4:**
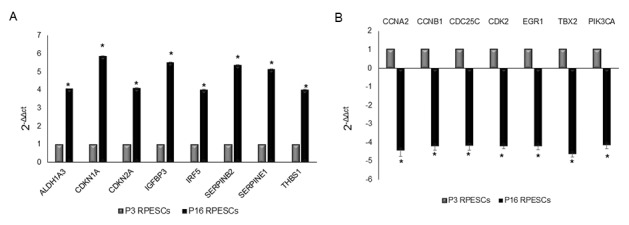
**Senescence-associated gene expression profile in senescent and young RPESCs.** PCR array performed to analyze the mRNA expression levels of senescence-associated genes in 3 senescent (P16) and 3 young (P3) RPESCs. Data are reported as fold change. A 4-fold difference was considered significant and only mRNAs with a ΔΔCt greater than 4 (**A**) or lower than -4 (**B**) are reported. *P = from 0.018 to 0.046.

Fifteen genes related to human cellular senescence were differentially expressed and showed a 4-fold change in senescent compared to young RPESCs: 8 genes, *ALDH1A3* (aldehyde dehydrogenase 1 family member A3), *CDKN1A* (cyclin-dependent kinase inhibitor 1A, p21), *CDKN2A* (cyclin-dependent kinase inhibitor 2A, P16INK4), IGFBP3 (insulin-like growth factor binding protein 3), *IRF5* (interferon regulatory factor 5), *SERPINB2* (serpin family B member 2, PAI2), *SERPINE1* (serpin family E member 1, PAI1), and *THBS1* (thrombospondin 1), were upregulated (Figure 4A), whereas 7 genes, *CCNA2* (cyclin A2), *CCNB1* (cyclin B1), *CDC25C* (cell division cycle 25C), *CDK2* (cyclin-dependent kinase 2), *EGR1* (early growth response 1), *TXB2* (T-box 2), and *PIK3CA* (phosphatidylinositol-4,5-bisphosphate 3-kinase catalytic subunit alpha), were downregulated (Figure 4B).

### p21 and p53 protein expression in young and senescent RPESCs

**Figure 5:**
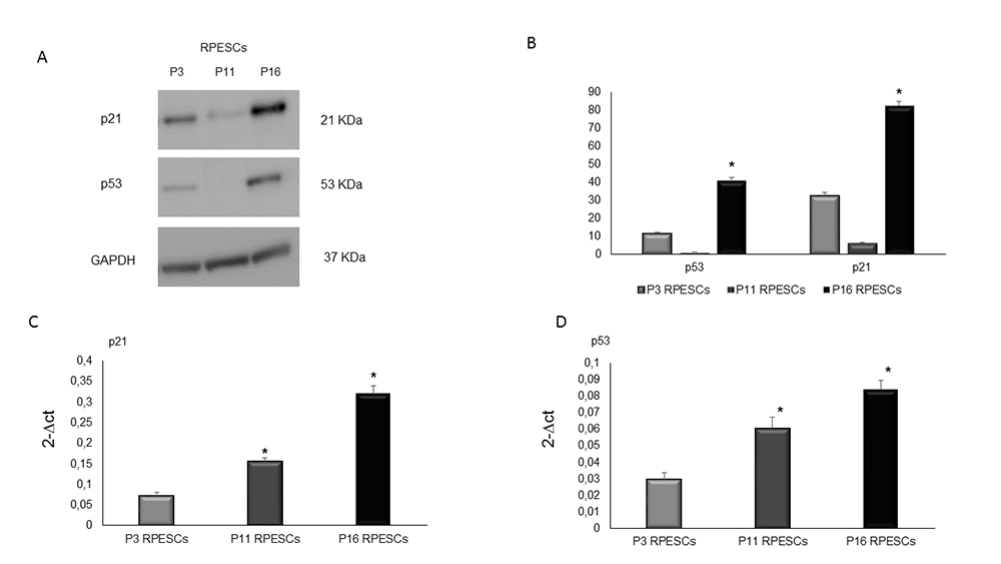
**Human p53 and p21 protein expression levels in senescent and young RPESCs.** p53 and p21 protein expression levels (**A**) Western blot analysis (**B**) and densitometric analysis of blots. Data are mean ± SD of 3 independent experiments. *P = from 0.022 to 0.046. (**C**) Relative expression levels of mRNA related to p21 and (**D**) p53 genes in young (P3), pre-senescent (P11) and senescent (P16) RPESCs cells. Data are mean ± SD of 3 independent experiments. *P = from 0.031 to 0.044.

Western blot analysis demonstrated that p21 and p53 protein were significantly upregulated in senescent compared to young RPESCs (Figure 5A) and that intermediate-passage cells (P11) were downregulated compared to both young and senescent RPESCs. The results of densitometric analysis and normalization to GAPDH expression (Figure 5B) were confirmed by real time PCR, which demonstrated that p21 and p53 mRNA was upregulated in senescent (P16) compared to young RPESCs (P3) (Figure 5C and D). In contrast, P11 RPESCs showed higher p21 and p53 mRNA and lower protein levels compared to young RPESCs (P3).

## DISCUSSION

Age-related inflammation is a major risk factor in the aging process. AMD is a highly common, irreversible cause of severe loss of vision among the elderly in developed countries [2]. It has a multifactorial etiology, where advanced age and genetic predisposition are the strongest risk factors [27,28]. Although the mechanisms involved in its pathogenesis are unclear, inflammatory pathways have been reported to play a key role in its development and progression, as in the case of other ARDs [29]. Recent data have lent support to the hypothesis that senescent cells accumulate in the aging primate RPE [30]. Senescent cells accumulate with aging in human tissues and organs, promoting ARD development and progression; cell exposure to recurrent or chronic stress, including oxidative stress, may also result in their accumulation [31,32]. Our group has recently characterized a stem cell-like population derived from the human RPE [25] as RPESCs (RPE progenitor cells). Notably, MSCs enter replicative senescence after a limited number of cell divisions, a fact that needs to be considered in experiments involving cell cultures, especially in investigations of regenerative medicine approaches. The effects of aging, senescence, and oxidative stress can induce loss of differentiation and proliferative potential in adult MSCs, including RPESCs [33]. Senescent cells acquire the SASP and release various proteins, including proinflammatory molecules, thus contributing to ARDs and the associated morbidity [34]. Critically, although human RPESCs have been identified as a stem-cell like population, they are unable to differentiate into mature RPE cells, replacing those that are lost due to AMD. In our previous study we isolated, cultured, and characterized RPESCs from the human RPE and investigated their differentiation potential [25]. In the present work, induction of replicative senescence in RPESCs resulted in reduced proliferative and multilineage differentiation potential, SASP acquisition, and release of inflammatory proteins that may be involved in AMD development and progression. Senescent RPESCs exhibited telomere length shortening and a characteristic large and flattened morphology. They also stained for SA β-gal and showed upregulation of p21 and p53 protein compared to young RPESCs. p21 and p53 are key components of the senescence machinery, playing a critical role as regulators of stem cell functions [35]. Senescent RPESCs expressed higher mRNA levels of senescence-associated genes compared to their young counterparts. These genes, which include *CDKN1A* (p21), *IGFBP3*, *SERPINE1*, and *SERPINB2*, have been implicated in the maintenance of replicative and stress-induced cellular senescence mechanisms [36,37]. In addition, *SERPINE1* (PAI1) is responsible for decreased extracellular matrix (ECM) degradation through inhibition of metalloprotease activation, mechanisms that may be involved in ECM accumulation in the RPE of AMD patients [38].

The irreversible growth arrest of senescent RPESCs was confirmed by the downregulation of the genes involved in cell cycle progression, including *CCNA2*, *CCNB1*, and *CDK2* [39,40]. The transcription factors *SOX2, KLF4,* and *c-MYC* have been reported to play a regulatory role in stem cell self-renewal, i.e. reprogramming. Interestingly, these stem-cell specific genes showed a similar expression level in senescent and young RPESCs, whereas the RPE-specific genes (*RPE65, MITF, OTX2, PAX6, CRALBP,* and *PEDF*) were downregulated in senescent RPESCs. These data suggest that despite their loss of differentiation potential during senescence, the reprogramming ability of these cells is preserved.

Senescent cells are metabolically active and release high concentrations of proinflammatory cytokines, chemokines, growth factors, and proteases into the culture medium [24,41]. Senescent RPESCs (P16) secreted higher IL-6, IL-12, IL-17, INF-γ, TNF-α, and TGFβ1 concentrations compared to young RPESCs. IFN-γ, TNF-α, and IL-17 are involved in Th1 and Th17 inflammation response pathways [42]. A recent study of the CD4+ T cell compartment in AMD patients has found that these cells play a proinflammatory role in an IFN-γ- and IL-17-dependent fashion [43]. Such proinflammatory cytokines are also likely to play a key role in AMD pathogenesis, and their effect may well be reinforced by senescent RPESCs.

Interestingly, proinflammatory cytokines may induce activation of the anti-oxidative stress response in mature RPE cells, as reported in a study where protective anti-oxidant pathways were activated in mature RPE cells treated with oxidative agents and cultured with peripheral blood mononuclear cell-conditioned medium or with IFN-γ/TNF-α [44]. Moreover, T-cell–derived proinflammatory cytokines were able to induce in mature RPE cells the production and secretion of multiple chemokines, which can affect the immune homeostasis in the retina [45]. Indeed, there is growing evidence for a role of the adaptive immune system in the pathogenesis of neovascular AMD. Several studies have addressed the crucial role of macrophages in the development of choroidal neovascularization [46–48] and of atrophic changes in the AMD retina [49], while an association has been described between AMD and systemic leukocyte activity [50].

Singh and co-workers have demonstrated that the age-related decrement in Th1 frequency seen in healthy controls is absent in AMD patients, since the percentage of CD4^+^ T-cells expressing CCR6 was significantly reduced in patients with non-exudative as well as exudative AMD [51]. There is also evidence that CCR2 expression in circulating monocytes may play a role in the development of neovascular AMD [52,46]. Furthermore, significantly accelerated T-cell differentiation and aging have been described in the CD8^+^ T-cell compartment of patients with neovascular AMD [53].

As regards the pro/anti-inflammatory phenotype of senescent RPESCs, the present study found a significant downregulation of the anti-inflammatory cytokines IL-4, IL-10, and IL-13 in these cells. An increased or similar expression of these cytokines has been reported in the serum and aqueous humor of AMD patients compared with controls [54,55], probably due to the acquisition by senescent RPESCs of a specific proinflammatory senescence-associated phenotype and to the downregulation of anti-inflammatory cytokines *in vitro*.

This study is preliminary and as such suffers from some limitations. Replicative senescence was induced *in vitro* in RPESCs isolated from the eye of a single healthy young donor. The examination of samples from aged AMD patients and healthy subjects is expected to provide insight into the senescent proinflammatory status of RPESCs in the elderly and into their role in disease pathogenesis. It would also be interesting to investigate the effects of oxidative agents on young and senescent RPESCs from the mature RPE in terms of induction of apoptosis or activation of anti-inflammatory anti-oxidant pathways.

Altogether, the present findings indicate that RPESCs can undergo replicative senescence, which affects their proliferation and differentiation ability. In addition, senescent RPESCs acquired the SASP, which probably compounds the inflammatory RPE microenvironment during AMD development and progression. A greater understanding of the role of RPESCs in AMD pathogenesis is needed to find means to control the disease and modulate its progression.

## MATERIALS AND METHODS

### RPESC culture

RPESCs were isolated, characterized, and cultured as described previously [25]. Cells were maintained in RPE medium [MEM-α modified medium containing 2 mM L-glutamine, penicillin/streptomycin (1:100), 1% Na-pyruvate, 10% fetal bovine serum], supplemented with taurine, hydrocortisone, triiodothyronine (THT), and N1 (all from Sigma-Aldrich, Milano, Italy). RPESCs were incubated at 37 °C in a 5% humidified CO_2_ incubator. The medium was replaced every 3 days.

Replicative senescence was induced by culturing cells up to the 18^th^ passage (P18). Viable cells were counted at each passage by trypan blue staining using an automated cell counter (Thermo Fisher, Milano, Italy). Population doublings (PDs) were determined as current PDs = last PDs+log2 (collected cell number / seeded cell number); cumulative PD (CPD) was calculated as the sum of PD changes, as described previously [56].

### Telomere length measurement

Telomere length was measured using the Relative Human Telomere Length quantification qPCR assay kit (ScienCell Research Laboratories, San Diego, CA, USA) according to the manufacturer’s protocol and a quantitative RT-PCR technique (Cawthon’s method [57]), as described previously [56].

### Senescence-associated β-galactosidase (SA β-gal) staining

β-gal staining was performed using the Senescence β-Galactosidase Staining kit (Cell Signaling Technology, Leiden, The Netherlands) according to the manufacturer’s protocol.

### Phalloidin staining

For phalloidin staining, young (P3), intermediate passage (P11) and senescent (P16) cells were seeded on chamber slides and cultured for 3 days in RPE medium. They were then fixed in 4% PFA for 30 min, blocked with bovine serum albumin (BSA), 2.5% in PBS for 30 min, and permeabilized with Triton X-100, 0.2% in BSA/PBS for 10 min at room temperature. Subsequently, cells were stained with TRITC-labeled phalloidin (Sigma Aldrich) according to the manufacturer’s protocol. Nuclear staining was obtained by applying Hoechst solution (Molecular Probes, Thermo Fisher, Milano, Italy) for 10 min. Slides were mounted with Vectashield (Vector Laboratories, Burlingame, CA, USA).

### Analysis of the secretory phenotype

The concentration of IL-4, IL-6, IL-10, IL-12, IL-13, INF-γ, TNF-α, and TGFβ1 was measured in the supernatant by an ELISA method (Multi-Analyte ELISArray kit, Qiagen; Affymetrix Ebiosciences, Vienna, Austria). Briefly, the supernatants were collected at the end of each passage before trypsinization, centrifuged, and stored at -20 °C. Optical density at 450 nm was determined using a microtiter plate reader (Multiskan Go, Thermo Scientific, Monza, Italy). Cytokine concentration was determined as pg/ml by comparing their absorbance to the antigen standards. Each experiment was performed three times.

### RNA isolation

Total RNA from P3 and P16 RPESCs was isolated using PerfectPure RNA Cell and Tissue Kit (5 PRIME, Hamburg, Germany). The concentration and purity of total RNA samples were determined using a NanoDrop One Spectrophotometer (NanoDrop Technologies Inc., Wilmington, DE, USA). RNA was stored at -80 °C until use. About 300 ng of total RNA extracted from both RPESC sets was reverse transcribed using the GoScript™ Reverse Transcription System (Promega, Milano Italy) according to the manufacturer’s protocol.

### RT-PCR analysis

Real-time PCR was performed with a Master Cycle (Eppendorf, Hamburg, Germany) apparatus using EVA Green PCR Master Mix (Bio-Rad, Milano, Italy) according to the manufacturer's instructions. Conditions were as follows: denaturation 98 °C for 2 min and 40 cycles of 98 °C for 60 s and 60 °C for 60 s. A melting stage was added at the end of amplification. There was no non-specific amplification as determined by the melting curve. All samples were tested in triplicate with the reference genes *β-actin* and *RPL30* for data normalization. Genes and related primer sequences (SOX2, KLF4, c-MYC, RPE65, CRALBP, PEDF, OTX2, MITF, p21 and p53) were as described previously [58]. The mRNA expression level of all tested genes was analyzed in young and senescent RPESCs with the 2^-ΔΔCt^ method: Δ (ΔCt) = ΔCt (senescent) − ΔCt (young). The relative expression values of the genes of interest are reported as mean ± standard deviation (SD) of three independent experiments.

### mRNA profiling

Total RNA from young (P3) and senescent (P16) RPESCs was isolated with the PerfectPure RNA Cell and Tissue Kit. cDNA synthesis was performed with RT^2^ First Strand Kit (Qiagen, Milano, Italy) and Cellular Senescence RT Profiler PCR Array (PAHS-050ZA; Qiagen). The average of glyceraldehyde 3-phosphate dehydrogenase (*GAPDH*) and ribosomal protein, large, P0 (*RPLP0*) was used for normalization. Only mRNAs with reads < 35 Ct in all three biological replicates were included in the analysis. After amplification, melting curves were acquired. mRNA expression was quantified with the 2^-ΔΔCt^ method. Relative gene expression values are reported as mean ± SD of three independent experiments.

### Protein extraction and immunoblotting

Total protein was extracted from young (P3) and senescent (P16) RPESCs (1 x 10^6^ cells/sample) as described previously [16], with some modifications. Membranes were incubated overnight with the primary p21 and p53 antibody (Santa Cruz Biotechnology, Santa Cruz, CA, USA) using GAPDH as the endogenous control, followed by incubation with the secondary antibody conjugated to horseradish peroxidase (Santa Cruz Biotechnology). Protein detection on the membrane was performed using the Clarity Western ECL Substrate Kit (Bio-Rad). The signals were captured with an Alliance Mini (UVITEC Cambridge, Cambridge, UK) system; the p21 and p53 bands were quantified with UVITEC software and their intensity was normalized by comparison to the housekeeping protein β-actin, used as a loading control. The intensity of each band was compared to the negative controls and any change was expressed as a percentage.

### Statistical analysis

Results are expressed as mean ± SD. Comparisons between groups were analyzed by paired-sample *t* test comparisons using SPSS 20.0 software. Significance was analyzed in data from at least three independent experiments. *P* values ≤ 0.05 were considered significant.
